# Surgery for Esotropia in a Case of Nanophthalmos

**DOI:** 10.7759/cureus.63728

**Published:** 2024-07-03

**Authors:** Takashi Negishi, Shintaro Nakao

**Affiliations:** 1 Department of Ophthalmology, Juntendo University Faculty of Medicine, Tokyo, JPN

**Keywords:** surgical effectiveness, preoperative assessment, esotropia, strabismus surgery, nanophthalmos

## Abstract

Nanophthalmos, characterized by an abnormally small ocular globe, presents significant challenges in the management of strabismus due to its unique anatomical constraints. This detailed case report highlights the intricacies and outcomes of strabismus surgery in a patient with nanophthalmos, providing valuable insights into the surgical considerations and adaptations required for this rare condition. The subject of this case, a young girl diagnosed with esotropia and high hyperopia, underwent unilateral medial rectus muscle recession in an attempt to correct her esotropia. Despite the careful surgical approach and postoperative management, a two-year follow-up revealed a limited response to the intervention, with improvements in visual acuity but continued presence of esotropia and lack of stereopsis development. This case sheds light on several key considerations in the surgical treatment of strabismus in nanophthalmos patients, including the potential for reduced surgical effectiveness due to the small globe size, the importance of accurate preoperative assessment, and the challenges in predicting surgical outcomes. Additionally, it discusses the implications of these findings for future surgical planning, the potential need for revision surgeries, and the broader research context, emphasizing the necessity for a deeper understanding of the biomechanical and anatomical particularities of nanophthalmos in the context of strabismus surgery. The report concludes with recommendations for improving surgical strategies and patient outcomes, advocating for more comprehensive studies and a tailored approach to treating strabismus in individuals with nanophthalmos.

## Introduction

Nanophthalmos is a rare and complex developmental disorder of the eye characterized by the presence of a small globe within the eye, which can lead to various ocular complications [1,2]. Despite the limited reports on strabismus surgery for nanophthalmos, there are concerns regarding overcorrection and consecutive exotropia [3]. In this case report, we present a patient with nanophthalmos and esotropia who exhibited a reduced response to a unilateral medial rectus muscle recession two years following surgery. This case highlights the challenges and potential complications associated with strabismus surgery in patients with nanophthalmos, and the need for further research and understanding of the condition to improve surgical outcomes.

## Case presentation

A 3-year-old girl presented at the Juntendo University Department of Ophthalmology with a history of esotropia since age 1. She had been diagnosed with esotropia with high hyperopia at a different hospital 1 month prior and had been wearing glasses for 2 weeks. She was born by normal vaginal delivery following a 39-week gestation after an uncomplicated pregnancy and weighed 2,956 g at birth. The patient’s familial history was unremarkable. 

**Figure 1:**
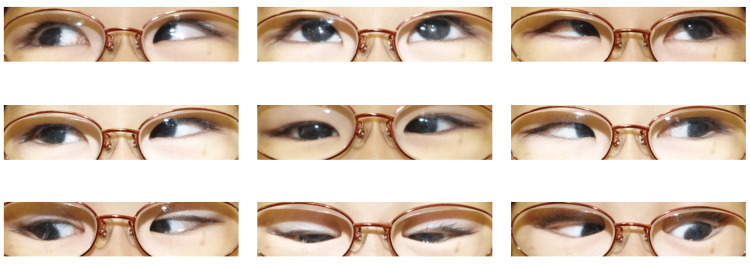
Nine gaze photos before surgery Even after wearing glasses, the patient shows esotropia.

Upon ophthalmological examination, her refraction was +14.50 -1.25 × 155 in the right eye and +16.25 -1.25 × 19 in the left without cycloplegics. The patient’s visual acuity was 0.15 in the right eye, and 0.1 in the left eye with a spectacle correction of +15.50 -1.50 × 160 (right) and +16.00 -1.00 × 020 (left). A dilated fundus examination showed no crowded optic discs and no abnormal folds. The alternate prism cover test revealed esotropia of 16Δ for distance fixation and 18Δ for near fixation with spectacle correction. A full range of eye movements was observed (Figure 1). Further, an evaluation of stereoacuity by the Titmus Fly test was negative.

Nine months later, the best-corrected visual acuity of the right eye was 0.5, while that of the left eye was 0.2. Consequently, patching of the right eye for 2-3 hours daily was initiated and continued.

When the patient reached the age of 6 years, her hyperopia remained unchanged, but her best-corrected visual acuity had improved bilaterally to 1.0. Further examination revealed esotropia of 25Δ for distance fixation and 30Δ for near fixation with spectacle correction. Axial lengths measured using the IOLMaster™ V.3.02 (Carl Zeiss Meditec GA, Jena, Germany) were 15.54mm and 15.52mm in the right and left eye, respectively. The accommodative convergence to accommodation ratio (AC/A ratio) was determined to be 3.33 using the far gradient method. 

**Figure 2:**
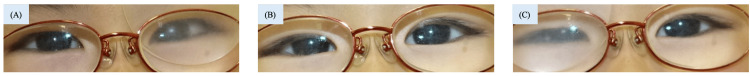
Eye positions 6 months after the surgery. Photo B seems to be orthophilia, but compared to photo C, small esotropia remained. A: Left eye covered B: Both eyes opened C: Right eye covered

**Figure 3:**
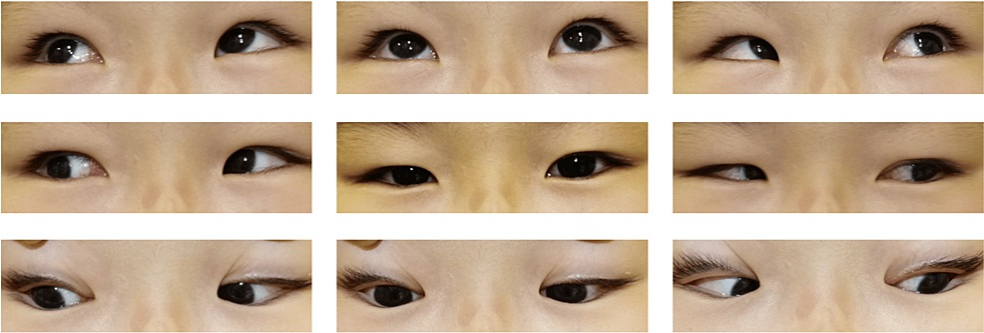
Nine gaze photos 6 months after the surgery without correction. Esotropia was reduced to 16 prism diopters.

Accordingly, a 6.0mm recession of the left medial rectus muscle was performed, achieving a reduction of esotropia to 16Δ for both distance and near fixation 3 months after the surgery. The best-corrected visual acuity of the left eye decreased to 0.8 after the surgery despite continued patching of the right eye for 2-3 hours daily. Six months after the surgery, her esotropia was still 14Δ for near and 18Δfor distance (Figure 2, 3). The patient suffered from juvenile idiopathic arthritis and began taking oral steroids. 

An ophthalmological examination 2 years after the surgery revealed esotropia of 18Δ for distance fixation and 18Δ for near fixation with spectacle correction.

## Discussion

Nanophthalmos, also referred to as simple microphthalmos, is a rare developmental ocular disorder characterized by the presence of a globe that is reduced in volume but is otherwise normal concerning internal organization and function [4]. Nanophthalmos is quite frequently associated with esotropia [3]. However, to date, no reports on strabismus surgery for nanophthalmos have been published, despite the several challenging issues associated with the surgery.

**Figure 4:**
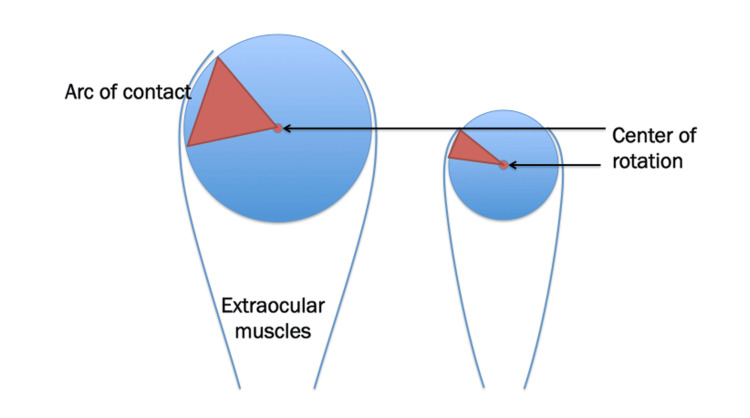
Conceptual diagram showing a change in the center of rotation as a result of a reduction in the diameter of the eyeball This is an original figure created by the authors. Small axial length = small arc of contact Small axial length = center of rotation differs from normal axial length. Strabismus surgery changes the position of the muscle attachment on the globe. For example, a 5mm recession produces a different reaction on the small eyeball compared to an eyeball of normal size.

The first concern regarding strabismus surgery for nanophthalmos is the possibility of different surgical outcomes as the result of normal axial length. A small diameter of the eyeball translates to a small arc of contact and a different center of rotation (Figure 4).

Further, a statistically significant inverse correlation was found between axial length and the surgical response of medial rectus recession for esotropic patients [5]. Supposing that the diameter of the eyeball is equivalent to the axial length (15.5mm), 6mm recession changes the insertion of the medial rectus to 119.1° toward the axis. Additionally, a different center of rotation might result from adduction insufficiency after an increased amount of nanophthalmos recession surgeries [6]. The second problem is consecutive exotropia [3]. The medial rectus muscle is the only rectus muscle that is not crossed to any other oblique muscles. Consequently, a slipped muscle might occur several decades after the surgery. The third problem is the timing of the surgery. Although a very early surgery corresponds to the possibility of good stereopsis, early surgical interventions are challenging because of the co-existence of an unstable angle of deviation, nanophthalmos, and ametropic amblyopia [3,7,8].

Şener et al. reported that there was no apparent need to decrease the surgical dose of the smaller axial length after considering the surgical results of five cases [3]. Kushner also concluded that the axial length had a minimal influence on the strabismus surgery compared to preoperative deviation [9]. However, our case had an extra short axial length compared to the above reports and might have led to unpredictable surgical results considering the biomechanics of strabismus surgery for a small eyeball. Thus, the amount of unilateral medial rectus muscle recession was reduced to minimize risks associated with postoperative overcorrection. Unfortunately, our patient experienced residual esotropia and no improvement of near stereopsis even though visual acuity improved. The deviation changed from 30Δ esotropia to 18Δ after a 6mm recession of the unilateral medial rectus muscle, which is usually expected with a 25Δ correction. As a result, the surgical outcome was insufficient after 2 years of observation. The movement of the eye is supported not only by extraocular muscles but also by a pulley connected to the orbital walls [10]. The orbit itself is very shallow in nanophthalmos [11]. We speculated that the recession surgery might have a reduced effect in nanophthalmos because the pulley might have a greater effect in a shallow orbit. Despite our concern regarding overcorrection, the current case also revealed a poor response to the recession surgery. Further, the amount of medial rectus muscle recession required for nanophthalmos might not be decreased, as indicated in previous case reports [3]. 

## Conclusions

This case report emphasizes the surgical challenges and unpredictable outcomes encountered in treating strabismus in patients with nanophthalmos. Despite tailored surgical modifications to account for the unique ocular anatomy, our patient showed a limited response, underscoring the complexity of achieving optimal results in such cases. The persistence of esotropia post-surgery highlights the necessity for individualized surgical strategies and the importance of understanding the intricate biomechanics associated with nanophthalmos. This case adds to the sparse literature on the subject and reinforces the need for ongoing research, improved surgical techniques, and better preoperative assessments to enhance outcomes for nanophthalmos patients undergoing strabismus surgery.
